# Supplementary material for: Larger models yield better results? Streamlined severity classification of ADHD-related concerns using BERT-based knowledge distillation
Source: PLoS One. 2025 Feb 6;20(2):e0315829. doi: 10.1371/journal.pone.0315829 (PMC11801592; doi:10.1371/journal.pone.0315829)
Supplement: Attachment — Submitted filename: Response To Reviewers.pdf [file pone.0315829.s002.pdf]

Manuscript ID: PONE-D-24-32612
Manuscript Title: Larger models yield better results? Streamlined severity classification of ADHD-related concerns using BERT-based knowledge distillation

Dr. Weiqiang (Albert) Jin, Ph.D.
Academic Editor
PLOS ONE

Dear Dr. Jin,

We would like to thank you and the respected reviewers for the time and effort spent reviewing our manuscript titled: **Larger models yield better results? Streamlined severity classification of ADHD-related concerns using BERT-based knowledge distillation** (PONE-D-24-32612). We appreciate the insightful comments and constructive feedback, which have been invaluable in improving the quality of our work.

In response to the reviewer's feedback, we have carefully revised the manuscript. Below, we provide detailed responses to the individual comments raised by the reviewers. We hope that the changes made to the manuscript have satisfactorily addressed all the concerns raised by the reviewers. We are confident that these revisions have significantly improved the clarity, quality, and scientific rigor of the manuscript.

Once again, we appreciate the constructive feedback and look forward to your favorable consideration of the revised version.

Sincerely,

Ahmed Akib Jawad Karim
On behalf of all co-authors

Additional requirements:

- **Question 1: Please ensure that your manuscript meets PLOS ONE's style requirements, including those for file naming. The PLOS ONE style templates can be found at**
  PLOS ONE Formatting Sample: Main Body
  PLOS ONE Formatting Sample: Title, Authors, Affiliations
  Response:
  Thank you for your guidance. All elements of the manuscript have been prepared according to PLOS ONE's style requirements. The following checks have been performed to ensure compliance:
  - The **Title**, **Main Body**, **Authors**, and **Affiliation** sections have been formatted according to PLOS ONE's style templates.
  - All figures, tables, and algorithms have been labeled and numbered consistently throughout the manuscript.
  - Each corresponding file is named to match the label used in the manuscript. For example, if a figure is referenced as "Figure 1" in the manuscript, the associated file is named `Figure1.png`. The same convention is applied to all tables (`TableX.ext`) and algorithms (`AlgorithmX.ext`).
  - The manuscript has been formatted according to the PLOS ONE templates, ensuring alignment with section headings, author affiliations, and references.

  We confirm that all submission standards have been adhered to. Please let us know if further adjustments are needed.
- **Question 2: Please note that PLOS ONE has specific guidelines on code sharing for submissions in which author-generated code underpins the findings in the manuscript. In these cases, we expect all author-generated code to be made available without restrictions upon publication of the work.**
  Please review our guidelines at ensure that your code is shared in a way that follows best practice and facilitates reproducibility and reuse.
  Response: We confirm that all author-generated code underpinning the findings in this manuscript is openly shared and available without restrictions. The code is hosted on GitHub, ensuring reproducibility, reuse, and compliance with PLOS ONE's guidelines on code sharing. The repository can be accessed at the following link:
  GitHub Repository: github.com/AkibCoding
  The repository includes comprehensive documentation and all scripts required to replicate the experiments described in this paper. For the convenience of researchers, installation instructions and dataset access information are also included.
- **Question 3: Please update your submission to use the PLOS LaTeX template. The template and more information on our requirements for LaTeX submissions can be found at http://journals.plos.org/plosone/s/latex.**

  Response:

Thank you for the reminder regarding the use of the PLOS LaTeX template. We acknowledge the importance of aligning with the journal's formatting requirements to ensure smooth processing and publication. We have updated our manuscript to use the official PLOS ONE LaTeX template, as requested. The structure, formatting, and styling of the manuscript now comply with the guidelines provided by PLOS ONE.
The revised submission incorporates:

- Title and Author Formatting: Adjusted to fit PLOS ONE's structure, including affiliations and corresponding author formatting.
- Section Headings: Updated according to the template's specifications.
- Figures and Tables: Aligned with the PLOS ONE formatting standards to ensure proper referencing and layout.
- Bibliography: Adapted to the PLOS ONE LaTeX reference style.

The LaTeX template and guidelines were followed from the source: `http://journals.plos.org/plosone/s/latex`.
We have also ensured that all required components, such as acknowledgments, conflict of interest statements, and data availability statements, are placed in the appropriate sections according to the PLOS ONE template guidelines.
The revised manuscript prepared using the PLOS ONE template, is included in this resubmission. Please let us know if further adjustments are needed.

- **Question 4: When completing the data availability statement of the submission form, you indicated that you will make your data available on acceptance. We strongly recommend all authors decide on a data sharing plan before acceptance, as the process can be lengthy and hold up publication timelines. Please note that, though access restrictions are acceptable now, your entire data will need to be made freely accessible if your manuscript is accepted for publication. This policy applies to all data except where public deposition would breach compliance with the protocol approved by your research ethics board. If you are unable to adhere to our open data policy, please kindly revise your statement to explain your reasoning and we will seek the editor's input on an exemption. Please be assured that, once you have provided your new statement, the assessment of your exemption will not hold up the peer review process.**
Response:
Thank you for your detailed guidance regarding the data availability policy. We confirm that all the data underlying the findings of our manuscript will be made freely accessible upon acceptance. We have prepared a comprehensive data-sharing plan to ensure compliance with PLOS ONE's open data policy, as outlined below:
1. GitHub Repository: The code, models, and documentation associated with our research are available in the following GitHub repository:

- `https://github.com/AkibCoding/Streamlined-ADHD-Severity-Level-Classification.git`

2. Hugging Face Repository: To facilitate model sharing and ease of access for the research community, the final distilled models (LastBERT) have also been uploaded to the Hugging Face platform:

– `https://huggingface.co/Peraboom/LastBERT`

3. Data Sharing Plan: Our research utilized both publicly available datasets and data collected during the study. As per the data policies:

– `https://www.kaggle.com/datasets/akibjawad/adhd-related-concerns`

Public datasets are appropriately cited and linked within the manuscript. Moreover, the processed, and derived data used for experiments will be included in the GitHub repository.

4. Access Restrictions and Compliance: Currently, no access restrictions are required for the data used in this research, as the datasets are anonymized and do not contain personally identifiable information. Thus, there are no ethical or legal barriers preventing public deposition.

5. Post-Acceptance Availability: Upon acceptance, we will finalize and freeze the contents of the above repositories, ensuring all scripts, models, and datasets are well-documented. The repositories will be permanently available, providing long-term access to the research artifacts.

This approach guarantees that all data and resources essential for reproducing our findings are accessible, aligning with PLOS ONE's data-sharing policy. Please let us know if further clarifications or additional steps are required.

- **Question 5: Please ensure that you refer to Figures 1 and 2 in your text as, if accepted, production will need this reference to link the reader to the figure.**

  Response: We are sorry for the inconvenience. We have referred to figures 1 and 2 in our text. (Page 7, Section 3.1.2, last paragraph. Page 10 Section 3.1.7, first paragraph)

- **Question 6: We note you have included a table to which you do not refer in the text of your manuscript. Please ensure that you refer to Tables 1 and 7 in your text; if accepted, production will need this reference to link the reader to the Table.**

  Response: Thank you very much for your attention to detail. Tables 1 and 7 were not referred to. It has now been referred to. Moreover, we have thoroughly checked that all tables from 1 to 7 are referred to in our text. (Page: 6 - Section: 3.1.2-Paragraph 2, Page: 13 - Section: 3.3.1 -Paragraph 1, Page: 18 - Section: 4.2 - Paragraph 1, Page: 23 - Section: 4.3.4 -Paragraph 1, Page: 23 - Section: 4.3.4 -Paragraph 1, Page: 24 - Section: 4.3.5 -Paragraph 1, Page: 25 - Section: 4.3.6 -Paragraph 1)

- **Question 7: Please include captions for your Supporting Information files at the end of your manuscript, and update any in-text citations to match accordingly. Please see our Supporting Information guidelines for more information: http://journals.plos.org/plosone/s/supporting-information.**

  Response: Currently we don't have any supporting information for our manuscript.

- **Question 8: Please review your reference list to ensure that it is complete and correct. If you have cited papers that have been retracted, please include the rationale for doing so in the manuscript text, or remove these references and replace them with relevant current references. Any changes to the reference list should be mentioned in the rebuttal letter that accompanies your revised manuscript. If you need to cite a retracted article, indicate the article's retracted status in the References list and also include a citation and full reference for the retraction notice.**

  Response:

  Thank you for your suggestion regarding the review and update of the reference list. We have carefully addressed this comment by ensuring that all references are complete, accurate, and relevant. In particular, we confirm the following updates:

  – Verification of Retractions: We have reviewed the reference list against retraction databases and official sources to confirm that the retracted papers have been removed and additionally, relevant studies have been added. If any of the cited references are retracted in the future, we will, update the reference list to reflect the retracted status. Then include a citation to the retraction notice alongside the original reference.

  – Addition of Recent Studies (2022–2024): As suggested by one of the respected reviewer, we have incorporated recent and relevant studies to strengthen our work. These include both knowledge distillation (KD) with BERT-related research and ADHD-specific studies employing NLP techniques:

    Knowledge Distillation and BERT-related research:

    * Kim, J., et al. (2022). Tutoring Helps Students Learn Better: Improving Knowledge Distillation for BERT with Tutor Network. EMNLP 2022. Abu Dhabi, UAE. DOI: https://doi.org/10.18653/v1/2022.emnlp-main.498.
    * Lin, J., et al. (2023). Pretrained Transformers for Text Ranking: BERT and Beyond. JAMIA, 31(4), 949–956. DOI: https://doi.org/10.1093/jamia/ocad013.
    * Kim, J., et al. (2023). Bat4RCT: A Suite of Benchmark Data and Baseline Methods for Text Classification of Randomized Controlled Trials. PLOS ONE, 18(3), e0283342. DOI: https://doi.org/10.1371/journal.pone.0283342.
    * Karim, A. A. J. (2024). Peraboom/LastBERT: Streamlined ADHD Severity Level Classification Using BERT-Based Knowledge Distillation. GitHub. Available: https://github.com/AkibCoding/Streamlined-ADHD-Severity-Level-C[illegible] git.

    ADHD and NLP-related research:

    * MedRxiv (2023). Brain-charting Autism and ADHD: Neurobiological Insights and Overlapping Traits. Available: https://www.medrxiv.org/content/10.1101/2023.06.12.23291071v1.
    * Kim, J., et al. (2024). LERCause: Deep Learning Approaches for Causal Sentence Identification from Nuclear Safety Reports. PLOS ONE, 19(8), e0308155. DOI: https://doi.org/10.1371/journal.pone.0308155.

- Accuracy of URLs and DOIs: We ensured that all URLs are up-to-date, accessible, and correctly formatted. For readability, we moved lengthy URLs to the footnotes or supporting information, where appropriate.
- Alignment with In-Text Citations: We verified that all in-text citations match the references accurately. We also updated any numbering inconsistencies and ensured compliance with the PLOS ONE referencing guidelines.
- References Integrity Check: We reviewed each reference for proper formatting, completeness, and relevance. As a result, our reference list now reflects the latest research relevant to our field, addressing knowledge distillation and NLP applications for ADHD-related studies.

These updates ensure that the reference list is accurate, complete, and aligned with the latest developments in the field. Please let us know if further changes or clarifications are required.

Response to Additional Editor's comments:

- **Comment: Based on the reviewers' feedback, we recommend you need minor revisions. The authors should focus on the following key areas: clearly justify the choice of the BERT-based model over others like GPT or Llama, and provide a comparative analysis of fine-tuning versus knowledge distillation. Address the need for recent references, improve formula numbering, clarify LastBERT's innovation, and enhance data interpretability in tables. Additionally, simplify the introduction and conclusion, improve image clarity, and consider a detailed discussion on limitations.**

  Response:

  We appreciate the editor's feedback and have made the following revisions accordingly:

  1. **Justification for BERT-Based Model**: We expanded the discussion to explain why BERT-based models were chosen over larger models like GPT and LLaMA. BERT's bidirectional nature and efficiency make it ideal for sentence-level tasks like classification, especially in resource-limited environments, unlike GPT or LLaMA, which are more resource-intensive. (Page: 7, section:3.1.2)

  2. **Fine-Tuning vs. Knowledge Distillation**: A comparative analysis has been added in the discussion section (page: 27, paragraph: 2) to contrast fine-tuning with knowledge distillation. Fine-tuning offers high performance on specific tasks but is resource-demanding. Distillation offers efficiency and is suitable for real-time, resource-constrained settings.

  3. **Recent References**: We incorporated relevant studies from 2022 to 2024, ensuring alignment with current advancements in the field.

  4. **Formula Numbering**: All formulas have been properly numbered for clarity. (Page- 7, 8, 9)

  5. **LastBERT Innovation**: The discussion now clearly outlines LastBERT's innovation, focusing on its reduced parameters and applicability in resource-constrained scenarios, without sacrificing much performance. (Page-5 Section: 3.1.1 and Page-7 Section: 3.1.2)

6. **Data Interpretability**: We revised Table 7 to improve clarity by removing irrelevant columns and highlighting key metrics like Matthews correlation and Spearman correlation where applicable. (Page-26)
7. **Simplified Introduction and Conclusion**: Both sections have been streamlined for focus and readability.
8. **Improved Image Clarity**: Visuals in Figures 7, 8, and 9 have been improved for better clarity, with magnified areas for important details. (Page- 20)
9. **Discussion on Limitations**: A more detailed discussion on LastBERT's limitations, including its performance on CoLA and STS-B, has been added. (Page 26)

Response to Reviewer #1's comments and questions:

- **Question 1: There are no recent studies and comparative models from 2022 to 2024 in the references. Why?**
  Response:
  Thank you for your valuable feedback. The absence of studies from 2022 to 2024 in the initial version of the manuscript can be attributed to the timeline of the research. The project began in mid-2022, with the knowledge distillation experiments concluding in early 2024. During the literature review phase, many recent studies were either unpublished or inaccessible. However, the manuscript has now been updated to reflect relevant studies from 2022 to 2024, ensuring alignment with the latest advancements in knowledge distillation (KD), BERT-based models, and ADHD-related NLP applications. Below is a summary of the newly added studies:
  - *Tutoring Helps Students Learn Better: Improving Knowledge Distillation for BERT with Tutor Network (2022).* Introduces the Tutor-KD framework, demonstrating how advanced KD techniques improve student models, aligning with the design of LastBERT.
  - *MLKD-BERT: Multi-Level Knowledge Distillation for Transformers (2022).* Explores multi-level KD approaches for efficient text classification, supporting the structure of LastBERT.
  - *Bat4RCT: Benchmark Data and Baseline Methods for Text Classification (2023).* Presents benchmark datasets for BERT-based models, validating the comparative advantages of KD over fine-tuning, as applied in LastBERT. (Mentioned by Reviewer 2)
  - *LERCause: Deep Learning Approaches for Causal Sentence Identification (2024).* Demonstrates the application of BERT-based models in NLP tasks, similar to LastBERT's use in ADHD-related text classification. (Mentioned by Reviewer 2)
  - *Brain-Charting Autism and ADHD: Neurobiological Insights and Overlapping Traits (2023).* Uses NLP and deep learning to analyze ADHD traits, adding relevance to LastBERT's application in mental health diagnostics.
  - *Biomedical Text Ranking and Classification Using Pretrained Transformers (2023).* Examines how transformer-based models perform in biomedical text tasks, supporting the goal of streamlining BERT models for resource-limited environments.

Regarding comparative models, we apologize for not incorporating recent comparative models. Recent models like LLaMA and GPT have now been incorporated into our related works section, further aligning with the latest advancements in the field. The revised manuscript includes these updates and clarifies the rationale behind our model selection. (page 7) The reason we chose BERT-based models is because of their proven efficiency across many NLP tasks, making them a solid baseline for knowledge distillation. While newer advanced current models like LLaMA or GPT offer state-of-the-art capabilities, their size, often reaching billions of parameters, makes them impractical for resource-limited platforms like Google Colab and Kaggle.
This challenge inspired the development of LastBERT, a smaller, efficient BERT-based model that maintains strong performance while being suitable for real-world applications with limited computational resources. By leveraging BERT Large and BERT Base, we ensure a balance between accuracy and efficiency.

- **Question 2: The formulas in the text are not numbered, such as (1), (2), etc.**
  Response: Thank you for your valuable feedback. The formulas in the text are now numbered. ((Page- 7, 8, 9))
- **Question 3: There are two periods at the end of the "Introduction" section.**
  Response: We apologize for the inconvenience. We have removed one of the periods. (Page- 3)
- **Question 4: Are there any relevant references on the hyperparameter settings of LastBERT?**
  Response:
  The hyperparameter settings for LastBERT were carefully chosen based on previous research and experimental tuning to ensure a balance between performance and efficiency. As described in our methodology section (Page 6, Paragraph 1), we optimized the model by adjusting the number of hidden layers, attention heads, and intermediate size, following recommendations from recent works on knowledge distillation, such as those by Jiao et al. (2020) and Sanh et al. (2019).
  We also adopted the AdamW optimizer, as proposed by Loshchilov and Hutter (2019), due to its ability to decouple weight decay from the gradient update. Further details about the training procedure, including batch sizes, learning rates, and the learning rate scheduler, are available in the methodology section (Page 9, paragraph 1). These hyperparameters were selected to align with practices outlined by Devlin et al. (2018) for BERT-based models, ensuring effective training and generalization. These configurations enabled LastBERT to perform well on GLUE benchmarks and ADHD severity classification tasks, demonstrating the effectiveness of our approach.
- **Question 5: In Table 7, the number of parameters of LastBERT is larger than that of MobileBERT. Why is the Matthews correlation coefficient on the CoLA dataset and the Spearman coefficient on the STS-B dataset so different from those of MobileBERT? In addition, the comparison model in Table 7 lacks some data support, and the comparison model needs to be expanded to increase the data interpretability of LastBERT.**

Response: Thank you for your thoughtful question. After re-running tests, we observed that the Matthews correlation coefficient (MCC) for CoLA improved from 0.11 to 0.17, highlighting an improvement in how LastBERT processes linguistic acceptability. For STS-B, a Spearman coefficient of 0.35 was achieved (before it was 0.34). These results, though modest, align with the use of WikiText-2 (2 Million words) during distillation, which lacks the extensive linguistic and semantic nuances necessary for these datasets. In comparison, MobileBERT leveraged a more advanced two-stage distillation method, incorporating embedding size compression and intermediate layer distillation, and was trained on significantly larger corpora like BooksCorpus (800 Million words) and English Wikipedia (2.5 Billion words). This allows MobileBERT to perform better in tasks like CoLA and STS-B, which require deeper linguistic and semantic understanding. Due to computational constraints, we selected WikiText-2, which was feasible for our study. Despite these limitations, LastBERT performed well on text classification, sentiment analysis, and paraphrase identification tasks, making it highly relevant for practical applications beyond linguistic-specific tasks. These limitations and proposed improvements are further elaborated in the discussion section (page 26, paragraph 1).
Additionally, Table 7 (Page 26) has been expanded to include results from BERT Base, BERT Large, and TinyBERT, providing a more comprehensive comparison of models. To improve data interpretability, we have retained only the most relevant metrics for each dataset. For MRPC and QQP datasets, the metric shown is the F1 score, which is a better indicator for tasks involving the classification of paraphrases. For SST-2 and MNLI datasets, accuracy is used to measure the model's performance in sentiment analysis and natural language inference. For CoLA, the Matthews correlation coefficient is shown, which reflects grammatical acceptability. For STS-B, the Spearman correlation is provided to assess sentence similarity.
This restructuring ensures clarity and focuses on the key performance aspects of LastBERT relative to other models. We believe these adjustments resolve the issue regarding data support and improve the presentation of our findings. We appreciate your feedback and believe this analysis provides valuable context for interpreting the results.

- **Question 6: In Table 6, Study 1-6 lacks annotations and the method names should be indicated. In addition, there are inconsistencies in the Dataset part of Table 6. Is the Accuracy indicator still meaningful for reference?**
  Response: We appreciate your thoughtful comments. Based on the feedback, we have made the following revisions to improve Table 6:
  1. *Annotations and Method Names:* In the revised version of Table 6, we have clearly indicated the method names for each study, including distinctions such as *NLP Techniques*, *Machine Learning*, and *Multimodal Neural Networks*, according to the methods described in the original studies. For our models, we have used *Knowledge Distillation and NLP Techniques* for LastBERT and *NLP Techniques* for DistilBERT and ClinicalBERT to accurately reflect the methodologies applied. These clarifications ensure consistency and provide transparency regarding the techniques employed. (Page: 25)

2. *Dataset Inconsistencies:* We have reviewed and aligned the dataset names across all studies to eliminate inconsistencies. For example, we ensured that datasets such as *ADHD-200* and *Reddit Mental Health* are used accurately and in alignment with the methods outlined in the corresponding studies. This ensures that all dataset references are correct and consistent. (Page: 25)

3. *Accuracy Indicator Relevance:* We acknowledge that some studies report *F1-score* instead of accuracy, which may introduce challenges in direct comparison. To address this, we have updated the table to display both accuracy and F1-score where available, with a "-" symbol indicating missing values. While accuracy remains a relevant indicator, we also emphasize that different metrics (e.g., F1-score vs. accuracy) should be considered in the context of each study's objectives and methodology. In our case, both accuracy and F1-score are presented to maintain transparency and allow for meaningful comparisons with other works in the field.

With these revisions, we believe the updated table now provides a clearer comparison of the methods, datasets, and metrics used in the referenced studies, ensuring transparency and meaningful interpretation. (Page: 25)

- **Question 7: Can you clarify the innovation of the model? It is not easy to understand from the text and model framework.**
  Response:
  Thank you for your valuable feedback. We appreciate the opportunity to clarify the innovation of LastBERT. The innovation lies in the following aspects:

  1. *Balanced Trade-off Between Performance and Efficiency:* LastBERT offers a new configuration by optimizing key elements such as the number of hidden layers and attention heads, achieving competitive performance while significantly reducing the number of parameters to only 29 million. This allows it to perform efficiently across a variety of NLP tasks, similar to BERT-base but with far fewer computational demands. (Page: 5-6, Section: 3.1.1, paragraph 2)

  2. *Accessibility for Researchers and Developers:* A core innovation is designing LastBERT to be fine-tuned and deployed on cost-free platforms like Google Colab and Kaggle, which have limited computational resources. While existing distilled models (e.g., DistilBERT) are also smaller, LastBERT explicitly focuses on balancing size, adaptability, and ease of use to democratize access to NLP tools. (Page: 12, Section: 3.2.7, paragraph 1)

  3. *Adaptability Across Multiple NLP Tasks:* Unlike many task-specific distilled models, LastBERT is designed to perform well across multiple domains—such as text classification, sentiment analysis, and paraphrase identification—while maintaining a small model size. For example, our model outshines even the BERT large model in the QQP dataset. This makes it a versatile, general-purpose model for diverse NLP applications. (Page: 25, table: 6)

  4. *Application of Knowledge Distillation with a Novel Focus:* LastBERT leverages a targeted knowledge distillation process, using BERT-large as the teacher model and refining the student architecture with insights from TinyBERT and DistilBERT. This approach ensures that LastBERT retains critical knowledge while offering a significantly lighter and faster model.

We have now revised the **Methodology** section on *(Page-5 Section: 3.1.1 and Page-6 Section: 3.1.2)*, including a clearer discussion of the innovative aspects of LastBERT. Additionally, we updated the framework diagram to better illustrate these points, ensuring readers can easily grasp the novelty of our contribution.

- **Suggestion 1: It is recommended to check the clarity of all images in the text, especially the small images such as Figures 7, 8, and 9. It is also recommended to use a magnifying glass frame to display the important areas of some images, such as Figure 4.**
  Response: Thank you very much for your valuable recommendation. We have checked the clarity of all images in the text, especially the small images of Figures 7,8 and 9. We have magnified the text which dictates which curve is for which parameter not only for 7,8,9 but from Figure 5 to 10. Lastly, for Figure 4, we have magnified the important area for better visibility and readability. (Page: 20)

- **Suggestion 2: The conclusion section is redundant. It is recommended to simplify the content and add a discussion section to fully analyze the shortcomings of the model and areas for improvement.**
  Response: We sincerely thank the reviewer for the insightful suggestion. In response, we have added the discussion section to thoroughly address the shortcomings of LastBERT. The discussion now also proposes areas for improvement, such as leveraging more suitable pretraining datasets and integrating advanced NLP techniques to enhance clinical applicability. Accordingly, the conclusion has been simplified to maintain focus and prevent redundancy, ensuring a concise summary of the study's key contributions. (Page: 26–28)

- **Suggestion 3: It is recommended that the URLs appearing in the references be placed in the footnotes of the corresponding pages of the text.**
  Response: Thank you very much for your recommendation, we have adjusted accordingly. (Page: 28–32)

- **Suggestion 4: The introduction of the dataset in Section 3 is too much, so it is recommended to simplify it.**
  Response: Thank you for the insightful suggestion. The introduction of the dataset in Section 3 has been revised to provide a more concise overview. Redundant details were removed, focusing only on the essential aspects relevant to the ADHD-related dataset and its preparation process. This ensures a smoother reading experience while retaining clarity and completeness. The updated content can be reviewed on **pages 13** for your feedback and further suggestions.

- **Suggestion 5: For the contribution part in the "Introduction" section, it is recommended not to emphasize the use of free computing resources, because many related researchers also complete their experiments and research work based on the free computing resource platform. It is recommended to mention it in the experimental environment setup part in Section 3.**
  Response:
  Thank you for the insightful suggestion. In accordance with your recommendation, we have revised the manuscript as follows:

1. **Revision in the Introduction:** We have removed the mention of free computing resources from the "Introduction" section to maintain a focused discussion on the key contributions of our study. (Page: 2)
2. **Addition to Methodology Section:** We have included the description of the computational environment, including the use of Google Colab (T4 GPU) and Kaggle Notebooks (P100 GPU), in a dedicated subsection titled "Computational Setup" under the Methodology section. This ensures that the computational environment is described appropriately without overemphasizing the use of free resources, aligning with your suggestion. (Page: 13, Section: 3.2.7)

We believe these changes address your concerns and improve the clarity and structure of the manuscript.

- **Suggestion 6: The last part of the Introduction section should summarize the main contents of the remaining sections.**
Response:
Thank you for the helpful suggestion. We have addressed this by adding a paragraph at the end of the **Introduction** section, which now summarizes the structure and contents of the remaining sections of the paper. Specifically, the new paragraph has been inserted on **Page 3, Paragraph 2**, ensuring a clear overview of the paper's structure. This addition improves the coherence and readability of the manuscript by guiding readers through the flow of the research.
- **Suggestion 7: Are the data in Table 5 redundant? The values of the macro average and weighted average are the same, so it is recommended to keep only one of them.**
Response: Thank you for your valuable feedback and it is true that it is redundant. We have now kept only one of them and removed the macro average portion. (Page: 24)

Response to Reviewer #2's comments:

- **Comment 1: This paper makes several contributions to the NLP and mental health diagnostics field. First, it demonstrates the effectiveness of knowledge distillation in creating a significantly smaller BERT-based model, LastBERT, which reduces parameters without compromising performance. Second, the model shows strong generalization, achieving high performance on the GLUE benchmark across various tasks. Third, it offers practical utility by applying the model to ADHD-related social media data, where it gained a commendable 85% across multiple evaluation metrics. This paper provides a valuable tool for mental health professionals, highlighting its potential in resource-constrained environments.**
  First, this paper should provide a more precise justification for choosing the BERT-based model, particularly concerning the study's specific objectives. It is essential to articulate the strengths of BERT compared to other large language models, such as GPT and Llama. This comparison could enhance the reader's understanding of why BERT is more suitable for the tasks addressed in the research. Including empirical evidence or relevant literature highlighting these advantages would strengthen the argument. Overall, a more detailed discussion of these aspects is essential for a comprehensive evaluation of the model selection in this paper.
  Response:
  Thank you for acknowledging our work and providing insightful feedback. We have carefully considered your suggestion to provide a more precise justification for selecting BERT-based models over other large language models (LLMs), such as GPT and LLaMA. Below is a summary of the key reasons aligned with the broader objectives of this research.

  1. *Alignment with Study Objectives:* Our primary goal is to develop *LastBERT*, a smaller and more efficient model usable across a wide range of NLP tasks—such as *text classification, sentiment analysis, paraphrase identification, and question answering*—making it adaptable beyond mental health applications. The model is designed to be easily fine-tuned on *cost-free computational platforms like Colab and Kaggle.*
  2. *Strengths of BERT:* BERT's *bidirectional contextual embeddings* make it highly effective for classification and sentence-level tasks, which aligns well with our focus. In contrast, models like GPT and LLaMA, while excellent for text generation, are *less specialized for fine-grained classification tasks.*
  3. *Resource Constraints and Practical Feasibility:* LLMs like GPT or LLaMA, with their *billions of parameters*, are computationally expensive, requiring high-end infrastructure that is *unavailable to many.* BERT-based models, by comparison, can be fine-tuned efficiently within the *limited resources on platforms like Google colab and kaggle notebook.* Our objective with LastBERT is to make NLP tools accessible to all developers and researchers, regardless of their access to infrastructure. *LastBERT's reduced size* enables fast fine-tuning, ensuring real-world challenges can be addressed efficiently using *cost-free resources.*

4. *Proven Framework for Distillation:* BERT's modular design and the success of pretrained variants like *DistilBERT* make it an ideal candidate for knowledge distillation. *LastBERT* leverages this framework to maintain high performance with half the parameters of DistilBERT, and one-fourth parameters of BERT Base, ensuring it is *both scientifically valid and pragmatically feasible.*

In summary, BERT-based models were chosen not only for their *performance and adaptability* but also for their *practical suitability within resource-constrained environments.* By developing LastBERT, we demonstrate that effective NLP solutions can be built and deployed using limited resources, aligning with the broader goal of *accessible and inclusive NLP tools.*
In response to your feedback, we have added a new subsection, "*Rationale for Model Selection,*" in the **Methodology** section on *Page 6, Section 3.1.2* and in related works section *Page 3, Paragraph 1.* This addition includes a comparison with other LLMs like GPT and LLaMA, along with supporting literature, to provide a more comprehensive discussion and align the research with scientific expectations.

- **Comment 2: Second, this paper should discuss the merits and limitations of employing a student model for knowledge distillation, particularly in light of existing research demonstrating promising performance from fine-tuning current BERT-based models with small datasets in various NLP tasks, as evidenced by the articles listed below.**

  – Lin, J., Nogueira, R., & Yates, A. (2020). Pretrained Transformers for Text Ranking: BERT and Beyond. arXiv preprint arXiv:2010.06467.

  – Kim, J., Kim, J., Lee, A., & Kim, J. (2023). Bat4RCT: A suite of benchmark data and baseline methods for text classification of randomized controlled trials. Plos one, 18(3), e0283342.

  – Kim, J., Kim, J., Lee, A., Kim, J., & Diesner, J. (2024). LERCause: Deep learning approaches for causal sentence identification from nuclear safety reports. Plos one, 19(8), e0308155.

  This context is crucial, as it highlights that substantial and efficient modeling can be achieved by fine-tuning the small dataset without the complexity of creating a student model. A comparative analysis between the efficiency of fine-tuning existing models with small datasets and the potential benefits of using a distilled model would provide valuable insights. Additionally, the discussion should address scenarios where the student model might offer advantages and any trade-offs involved in this approach. In conclusion, more thoroughly exploring these aspects will enhance the paper's contribution to the field.

  Response: Thank you for your valuable feedback regarding the comparison between knowledge distillation and fine-tuning pre-trained BERT-based models on small datasets. To clarify, this study does not exclusively apply knowledge distillation to the ADHD classification task. Instead, knowledge distillation was employed to create a lightweight and versatile model, LastBERT, capable of handling various NLP tasks efficiently, with ADHD classification serving as one real-world application example. The model can be used for any language-related tasks.

This comparison is indeed critical, as fine-tuning has proven effective in various NLP tasks, as demonstrated in works by Lin et al. (2020) and Kim et al. (2023, 2024). In this study, we chose knowledge distillation for its ability to generate lightweight models like LastBERT, which offers reduced computational complexity and faster inference times, making it suitable for resource-limited environments where fine-tuning larger models may not be feasible. While fine-tuning has shown state-of-the-art performance on smaller, domain-specific datasets, it demands significant computational resources during training and inference, which can be limiting.

Nevertheless, LastBERT, developed through knowledge distillation, remains flexible and can also benefit from fine-tuning. Researchers can fine-tune LastBERT on domain-specific datasets, combining its lightweight architecture with the performance gains of task-specific adaptation. This dual capability offers efficiency from distillation and enhanced task performance from fine-tuning, providing a valuable solution in resource-constrained environments. The trade-off between fine-tuning and distillation is thus centered on resource demands: fine-tuning excels in performance but is computationally intensive, while distillation balances efficiency and accuracy.

As noted in the discussion (page 26), the limitations in LastBERT's performance on tasks like CoLA and STS-B can be attributed to the smaller pretraining dataset (WikiText-2) used in this study. Multi-stage distillation strategies, such as those employed by MobileBERT, could be explored further to improve LastBERT's performance in future work.

In response to your suggestion, I have added a comparative analysis in the discussion section, highlighting the resource trade-offs and use-case scenarios where each approach excels. This addition clarifies the merits and limitations of both fine-tuning and distillation-based methods.

Once again, thank you for your insightful suggestions, which have been addressed in the revised discussion.
